# Influence of malting procedure on the isoflavonoid content of soybeans

**DOI:** 10.1038/s41598-024-57914-1

**Published:** 2024-03-26

**Authors:** Alan Gasiński, Dawid Mikulski, Grzegorz Kłosowski, Joanna Kawa-Rygielska

**Affiliations:** 1 Department of Fermentation and Cereals Technology, Wrocław University of Environmental and Life Sciences, Chełmońskiego 37, 51-630 Wrocław, Poland; 2 Department of Biotechnology, Kazimierz Wielki University, K. J. Poniatowskiego 12, 85-671 Bydgoszcz, Poland

**Keywords:** Soybean seeds, Malting, Isoflavonoids, Germination, Plant sciences, Chemistry, Nutrition

## Abstract

The goal of this study was to analyse, whether malting technique (consisting of seed hydration, germination and drying) can be used to modify concentration of various isoflavonoids in soybean seeds. Seeds of three soybean varieties were germinated by different lengths of time (from 24 to 120 h) and dried by two different methods, typically used to produce so-called ‘light’ and ‘caramel’ malts. It was determined, that malting decreases concentration of 7-O-β-D-glucosides such as daidzin, genisitin and glycitin, while at the same time increasing concentration of aglycones (daidzein, genistein and glycitein). Increasing time of the germination period increased concentration of aglycones. ‘Caramel’ type malts were characterised with higher concentration of most of the isoflavonoids (daidzin, daidzein, genistin, genistein and glycitein) than ‘light’ type malts. Results of this study suggest that soybean malts can be an interesting substrate in the production of various food products with increased aglycone content.

## Introduction

Soybeans (*Glycine max*) are a legume originating from Asia, but consumed worldwide nowadays. Their main advantage is high protein and fat content, which makes them nutritious and calorie-dense food, which can be used as a meat substitute. Soybeans are also rich in various phenolic compounds and numerous studies have demonstrated that consumption of this legume can significantly decrease risk of various diseases such as cancer, atherosclerosis or cardiovascular disease^1,2^. One of the interesting classes of phenolic compounds present in the soybean seeds are isoflavonoids. Main isoflavonoids found in majority of soybean seeds are aglycones daidzein, genistein and glycitein and their 7-O-β-glucosides, dadzin, genistin and glycitin^3^. Many health-promoting benefits of these compounds were proven in the past^4,5^. Consumption of isoflavonoids can be beneficial to the humans and animals alike, as various authors have proven their estrogenic, aniangiogenic, antioxidant and anticancer activities as a part of the human diet as well as cattle feed^6^. This suggests that analysing various methods of increasing the isoflavonoid content in the soybean products might be interesting scope of study. One of the latest studies about isoflavone content, analysing, among others, concentration of isoflavonoids in soybean seeds have shown, that soybean sprouts were characterised with greater concentration of these compounds than dry, non-sprouted soybean seeds^7^. Unfortunately, food sprouts are rather labile food products, which require refrigeration and are prone to microbial contamination^8^. However, malting is a food processing procedure, which combines seed hydration, germination and finally, dries the germinated seeds to produce stable products which is easy in transporting and processing^9^. Malts are mainly produced from cereal grains (primarily barley), but in recent years, this technique was applied as a method to modify and improve various characteristics of legume seeds, such as lentils, common beans, mung beans or chickpeas^10–13^. The results in changes of the composition of various legume seed during the malting process can vary significantly depending on the legume species, however. For example, malted common beans and lentils are characterised with reduced concentration of phytic acid, while malting does not change content of this compound in chickpeas^14^. However, variety of effects seems to occur in large number of species. For example, concentration of so-called raffinose-family oligosaccharides (raffinose, stachyose and verbascose) decreases after hydration and germination of legume seeds in lentils, beans, cowpeas and chickpeas^11,15,16^. Soybean malting has also been analysed in the past by^17,18^, but not in typical malting technology, as the^17^ used various organic growth promoters during malting and Simons et al. introduced *Rhizopus* cultures during the germination of soybeans^17,18^. Barnes et al. describes, that various methods of food processing, especially fermentation, can influence concentration of isoflavonoids or different form of isoflavonoids in food products^19^. Traditional method of soybean processing, such as production of soybean drinks (soymilk), tofu, sufu (traditional, fermented tofu), miso, natto, soy sauce, tempeh and douchi influence concentration of isoflavonoids as well as contribution of particular groups of isoflavonoids to the total isoflavonoid content^20^. During the production of soybean drinks, concentration of aglycones increased, while concentration of isoflavonoid glycosides decreased due to the presence and activity of endo-1,6-β-glucosidases^21^. Production of tofu, however, reduces concentration of isoflavonoids, albeit it was found that use of calcium chloride, magnesium chloride, calcium sulphate, calcium lactate, calcium acetate or acetic acid solutions as a coagulant during production of tofu can help in the retention of some of the isoflavonoids^21,22^. During production of sufu (fermented tofu) isoflavonoid content decreases, but changes in the amount of particular isoflavone isomers (aglycones) can be detected due to the activity of β-glucosidase^23^. Similar effects due to the same reasons occur in soy natto, miso and tempeh^24–26^. However, one of the most popular fermented soy products, soy sauce is characterised with low concentration of isoflavonoids which is most probably due to the fact of a prolonged fermentation process needed for the production of soy sauce (typically, at least 6 months)^27,28^. These results suggest that malting technique might be a viable method in producing soybean food products with increased isoflavonoid content. As the malts are more viable substrate for fermentation than unmalted grains (due to increased amount of FAN, reducing sugars,and greater enzymatic activity and lower concentration of anti-nutritional factors) malted soybean could possibly be used as a novel substrate for production of fermented soybean products (such as soy sauce, miso, fermented tofu, natto or tempeh) with increased concentration of health-promoting isoflavonoids. The goal of this study was the assessment, whether it is possible to produce soybean malts from soybeans varieties, which are currently cultivated in cooler regions of the globe, not typical for soybean cultivation, such as Poland and if malting will have a significant influence on the composition of isoflavonoids in the acquired product. Subsequent goal of analyses performed in this study aimed at determination, whether factors such as length of germination, different soybean variety and type of malt produced (which results from the different type of drying used) have an crucial impact on the composition of isoflavonoids. Three of the chosen soybean varieties, five different lengths of soybean germination (24 h, 48 h, 72 h, 96 h and 120 h) and two types of drying (typical for ‘light’ malt and typical for ‘caramel’ malt) allowed for the production of thirty different malt types.

## Materials and methods

### Materials

#### Raw material

Plant material used in this study were seeds of three soybean (*Glycine max*) varieties grown in Poland: Abaca (ABAC), Abelina (ABEL) and Aurelina (AUR). Soybean seeds were acquired from the seed centre Saatbau (Środa Śląska, Poland) and were harvested in the year 2022. All procedures concerning use of the plant seeds were conducted in accordance to the institutional, national and international guidelines and legislation.

#### Reagents and standards

Reagents used in this study were acetonitrile (99%), formic acid (99%) (Honeywell, Charlotte, NC, USA) and sodium hypochlorite (15%) (Chempur, Piekary Śląskie, Poland).

Standards used in this study were daidzin (98.3% purity), daidzein (98% purity), genistein (99% purity), genistin (95.5% purity), glycitin (99% purity), glycitein (99% purity), suitable for HPLC analyses, purchased from the Sigma-Aldrich company (Saint Louis, MO, USA).

### Methods

#### Analysis of moisture content of the soybean seeds and soybean malts

Moisture of the unmalted soybean seeds and malts produced throughout the study was performed using MT Moisture Analyser (Brabender, Duisburg, Germany). Each sample was analysed in triplicate.

#### Malting procedure

Fifty gram portions of soybeans were weighed and transferred to perforated, stainless steel malting containers (20 containers for each of the soybean varieties), which were previously disinfected by drying in the UF110 Plus dryer (Memmert GmbH + Co, Schwabach, Germany) for 2 h at 200 °C and then cooled to room temperature. Containers filled with known mass of soybeans were then weighed (the container filled with soybean seeds will be from now on mentioned as the ‘malting kit’). Changes in the moisture content of the seeds during the first step of the malting process (steeping) were calculated based on the changing weight of the malting kit, assuming, that the increase in weight of the kit is equal to the weight of water adsorbed by the seeds. Steeping was executed in the water–air steeping cycle. At the start of the process, malting kits were submerged in the 1.5% sodium hypochlorite solution for 10 min to surface sterilise the seeds. Malting kits were then removed from the sodium hypochlorite solution and immediately washed three times with distilled water. After this process, malting kits were submerged in tap water (disinfected previously by boiling and cooled) at temperature of 18 °C for 6 h, then transferred to the KK 240 Smart Pro (PolEko Aparatura, Wodzisław Śląski) germination chamber (with humidity set at 90% relative humidity and temperature set at 18 °C) for 17 h; then submerged another time in fresh, disinfected tap water (18 °C) for 5 h. After each step, malting kits were weighed to determine changing moisture content of the soybean seeds. At the end of the steeping process, the moisture content of the soybean seeds was equal to 58% (± 0.5%). Soybean seeds were germinated in the germination chamber with the temperature set at 18 °C and relative humidity 90%. Every consecutive 24 h of germination, up to 120 h, 4 malting kits with each of the soybean variety seeds were removed and dried in the UF110 Plus dryer in the following conditions:

- two of the containers were dried in the temperature of 50 °C for 23 h to produce ‘light’ or ‘Pilsener’ malts
- two of the containers were dried in the changing temperature program: 65 °C for 4 h; increase to 80 °C (0.5 h, at a rate of 0.5 °C per min); 80 °C (6.5 h); increase to 90 °C (0.5 h, at a rate of 0.33 °C per min); 90 °C (6 h); increase to 110 °C (0.5 h, at a rate of 0.66 °C per min); 110 °C (5 h). During the first 4 h, flap of the dryer was closed to retain moisture inside of the dryer. This method was used to produce so-called ‘caramel’ malts^10,29^.

This malting procedure resulted in production of 30 different soybean malt samples:

- Abaca soybean malts dried by method ‘a)’, germinated for 24 h, 48 h, 72 h, 96 h and 120 h (ABAC1, ABAC2, ABAC3, ABAC4, ABAC5)
- Abaca soybean malts dried by method ‘b)’, germinated for 24 h, 48 h, 72 h, 96 h and 120 h (ABAC1-C, ABAC2-C, ABAC3-C, ABAC4-C, ABAC5-C)
- Abelina soybean malts dried by method ‘a)’, germinated for 24 h, 48 h, 72 h, 96 h and 120 h (ABEL1, ABEL2, ABEL3, ABEL4, ABEL5)
- Abelina soybean malts dried by method ‘b)’, germinated for 24 h, 48 h, 72 h, 96 h and 120 h (ABEL1-C, ABEL2-C, ABEL3-C, ABEL4-C, ABEL5-C)
- Aurelina soybean malts dried by method ‘a)’, germinated for 24 h, 48 h, 72 h, 96 h and 120 h (AUR1, AUR2, AUR3, AUR4, AUR5)
- Aurelina soybean malts dried by method ‘b)’, germinated for 24 h, 48 h, 72 h, 96 h and 120 h (AUR1-C, AUR2-C, AUR3-C, AUR4-C, AUR5-C)

Malts of each type from two separate containers were mixed together transferred to tightly closed containers, to prevent moisture absorption during cooling period. Malts, as well as unmalted soybeans, were ground with the use of Bühler Miag disc mill DLFU (Bühler, Uzwil, Switzerland), with the gap between the discs set at 0.2 mm.

#### Extraction of soy isoflavonoids

One gram of ground soybean malt or soybean sample was transferred to the 50 cm^3^ falcon tube. Ten cm^3^ of 70% acetonitrile was added to the tube. Tube was then vortexed for 30 s and then transferred to the ultrasonic bath and sonicated for 30 min. (20 °C). Tube was then vortexed one more time for 30 s and centrifuged for 10 min (6000 rpm). Supernatant was filtered through 0.45 µm syringe filter to the chromatographic vial. From each of the malt/seed types, three extracts were produced, which resulted in three vials with isoflavonoid extract per malt^30^.

#### Chromatographic analysis

Isoflavonoid extracts were analysed using high performance liquid chromatography. Chromatographic analyzes were performed using Agilent Technologies model 1220 (Agilent Technologies, Santa Clara, CA, USA) apparatus equipped with a thermostat and diode array detector (DAD). The separation was performed using Poroshell 120 EC-C18 (4.6 × 250 mm, 2.7 µm) (Agilent Technologies) column. Gradient elution was used throughout the analysis. Elution program was as follows:

- 0–15 min: 90% eluent A (0.1% formic acid); 10% eluent B (acetonitrile);
- 15–18 min: 50% eluent A (0.1% formic acid); 50% eluent B (acetonitrile);
- 18–25 min: 10% eluent A (0.1% formic acid); 90% eluent B (acetonitrile);

Injection volume was 1 mm^3^ and flow through column was equal to 1 cm^3^ per min. Detection was performed at the wavelength of 260 nm. Quantitation of the isoflavonoids was performed using ESTD method with calibration curves with the following LOQ: 1.14 µg of daidzin per cm^3^ of the extract; 1.40 µg of daidzein per cm^3^; 4.50 µg of genistin per cm^3^; 1.46 µg of genistein per cm^3^; 4.32 µg of glycitin per cm^3^; 1.25 µg of glycitein per cm^3^ and LOD equal to: 0.28 µg of daidzin per cm^3^ of the extract; 0.46 µg of daidzein per cm^3^; 1.48 µg of genistin per cm^3^; 0.34 µg of genistein per cm^3^; 1.26 µg of glycitin per cm^3^; 0.34 µg of glycitein per cm^3^^31^.

### Data analysis

Results of analysis of the isoflavonoid content were statistically analysed in the Statistica 13 program from Statsoft (Tulsa, OK, USA) with three-way ANOVA (using three factors: seed variety, germination time and drying type) (α = 0.05). Tukey test was used to determine homogenous groups. Principal component analysis (PCA) was conducted to compare the content of isoflavonoids in the soybeans and soybean malts. The single-linkage agglomerative method was used, employing the Euclidean measure to determine the distance between each isoflavonoid content and soybean/soybean malt sample.

## Results and discussion

**Figure 1:**
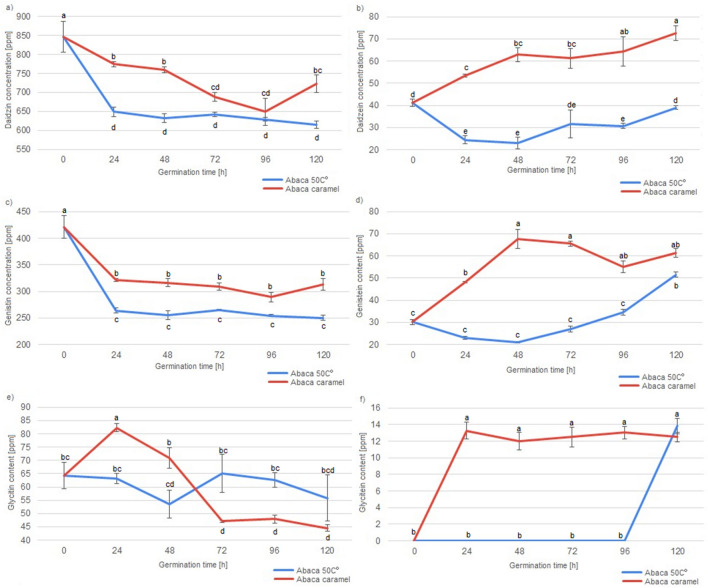
Concentration of various isoflavonoid in Abaca soybeans and Abaca soybean malts during the course of malting. Different letters present various homogenous groups (Tukey test, α = 0.05) in the concentration of particular isoflavonoid.

**Figure 2:**
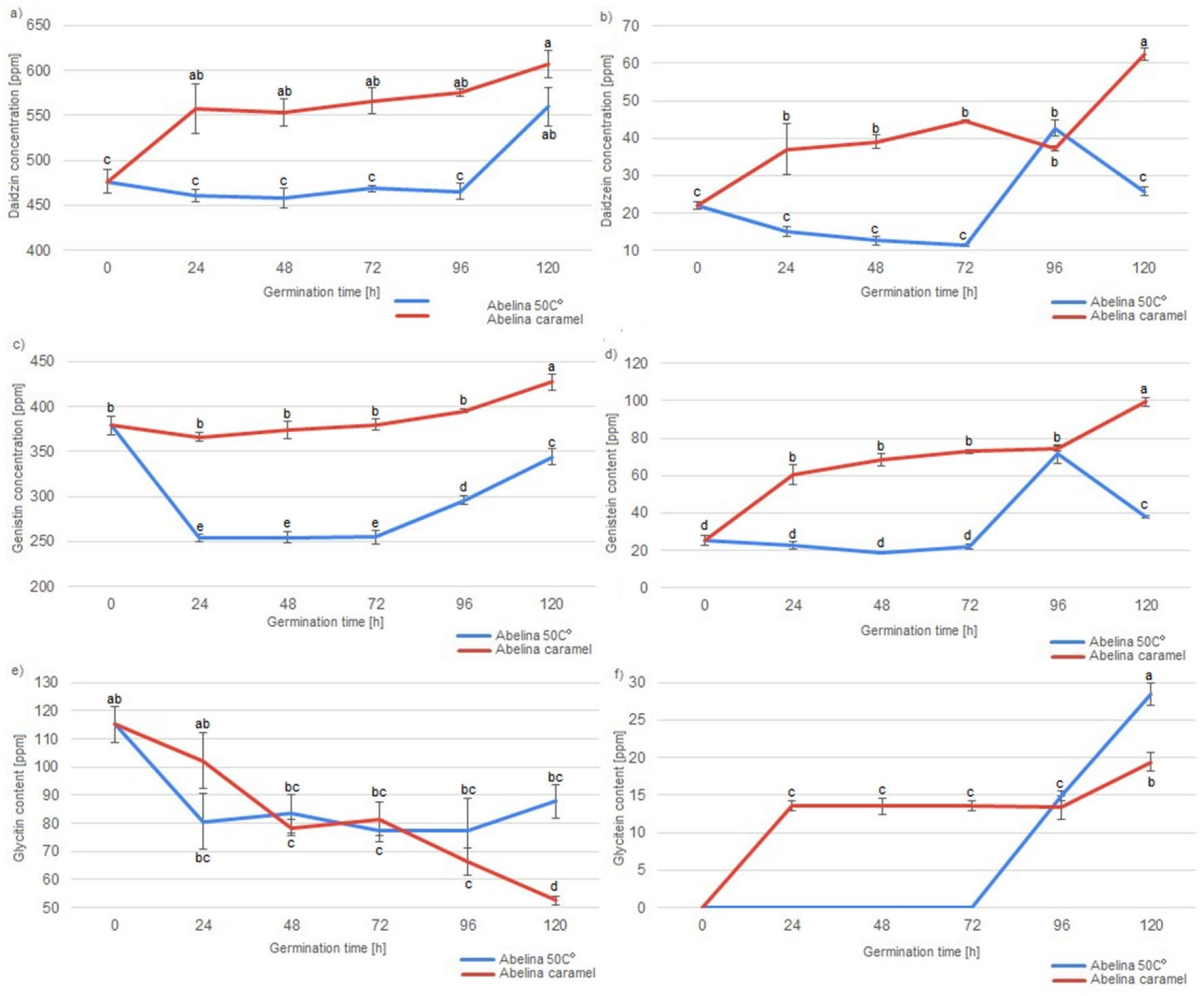
Concentration of various isoflavonoids in Abelina soybeans and Abelina soybean malts during the course of malting. Different letters present various homogenous groups (Tukey test, α = 0.05) in the concentration of particular isoflavonoid.

**Figure 3:**
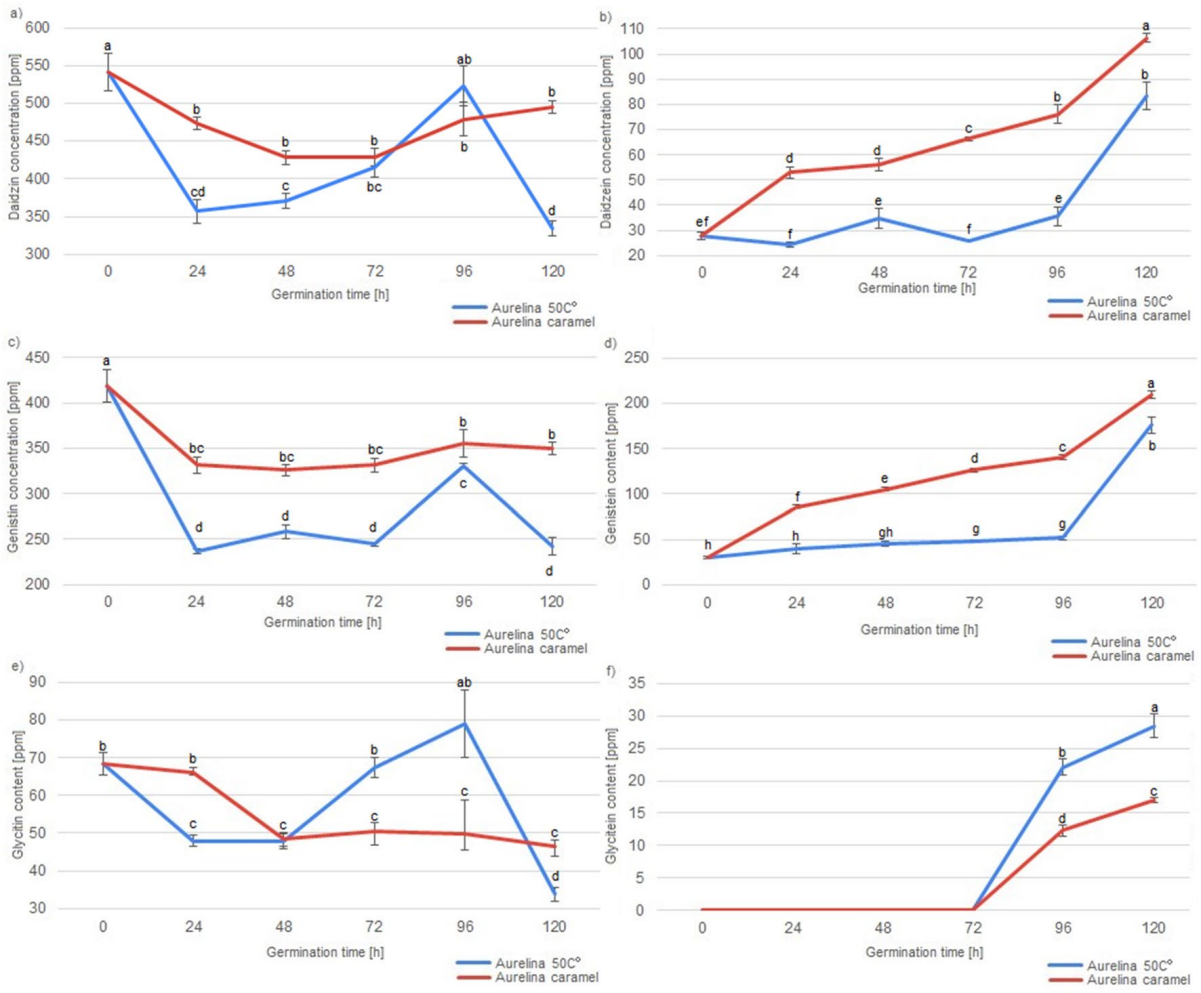
Concentration of various isoflavonoids in Aurelina soybeans and Aurelina soybean malts during the course of malting. Different letters present various homogenous groups (Tukey test, α = 0.05) in the concentration of particular isoflavonoid.

Total concentration of daidzin, daidzein, genistin, genistein, glycitin and glycitein in each of the soybean and soybean malt samples analysed throughout the study are shown in the Figs. 1, 2 and 3.

Table 1 shows concentration of isoflavonoids in the soybeans and soybean malts depending on the soybean variety (Abaca, Abelina and Aurelina), germination time (0 h for soybeans, which were not malted and 24 h, 48 h, 72 h, 96 h and 120 h for soybean malts) and drying procedure (continuous drying at 50 °C or type of drying used to produce caramel malts).

**Table 1:** Concentration of isoflavonoids in soybean seeds and soybean malts.

	Daidzin	Daidzein	Genistin	Genistein	Glycitin	Glycitein
	[ppm]					
Soybean variety	Concentration of isoflavonoids in soybeans and soybean malts of different varieties^1^					
Abaca	704.94 ± 84.88 a	45.48 ± 16.80 a	306.78 ± 58.81 b	42.96 ± 16.78 b	60.14 ± 11.52 a	6.43 ± 1.57 b
Abelina	518.55 ± 55.54 b	31.04 ± 15.13 b	342.03 ± 59.78 a	49.96 ± 26.80 b	84.89 ± 19.16 b	9.31 ± 3.08 a
Aurelina	449.23 ± 71.29 c	51.47 ± 26.15 a	320.33 ± 52.75 b	90.75 ± 48.99 a	56.16 ± 13.28 a	6.66 ± 1.22 b
Germination time	Concentration of isoflavonoids in soybeans and soybean malts germinated by different amount of time^2^					
Non-germinated	621.62 ± 128.94 A	30.37 ± 8.35 C	406.32 ± 26.56 A	28.72 ± 2.94 D	82.60 ± 24.26 A	n.d
24 h	545.51 ± 110.97 B	34.56 ± 15.41 C	295.58 ± 38.56 C	46.72 ± 22.90 C	73.73 ± 18.73 AB	4.483 ± 1.545 C
48 h	533.86 ± 106.52 B	38.09 ± 18.15 CB	297.58 ± 47.14 C	54.42 ± 31.03 BC	63.77 ± 15.45 BC	4.261 ± 1.249 C
72 h	535.19 ± 98.34 B	40.22 ± 20.13 CB	297.58 ± 49.60 C	60.28 ± 35.69 BC	64.87 ± 13.88 BC	4.344 ± 1.355 C
96 h	553.51 ± 75.25 B	47.79 ± 17.47 B	320.04 ± 48.27 B	71.34 ± 34.73 B	63.88 ± 14.18 BC	12.628 ± 6.797 B
120 h	555.74 ± 104.74 B	64.94 ± 27.75 A	321.18 ± 55.73 B	105.86 ± 46.79 A	53.53 ± 17.94 C	19.944 ± 6.709 A
Drying procedure	Concentration of isoflavonoids in soybean malts dried using different procedures^3^					
‘50 °C’	524.83 ± 117.56 **	30.67 ± 15.89 **	290.18 ± 50.71 **	43.22 ± 25.45 **	68.40 ± 19.02 *	5.99 ± 2.33 **
‘Caramel’	590.32 ± 124.31 *	54.66 ± 19.84 *	355.91 ± 42.28 *	79.22 ± 24.12 *	65.73 ± 20.27 *	9.23 ± 3.85 *

^1^Values are expressed as a mean (n = 33) ± standard deviation. Various small letters (a, b, c) indicate homogenous groups according to the variable ‘variety’ according to the Tukey test (α = 0.05).

^2^Values are expressed as a mean (n = 18) ± standard deviation. Various capital letters (A, B, C) indicate homogenous groups according to the variable ‘germination time’ according to the Tukey test (α = 0.05).

^3^Values are expressed as a mean (n = 45) ± standard deviation. Various number of asterisks (* or **) indicate homogenous groups according to the variable ‘drying procedure’ according to the Tukey test (α = 0.05). Data for unmalted soybeans seeds was not used in this comparison.

‘N.d.’ indicates that compound was not detected.

**Figure 4:**
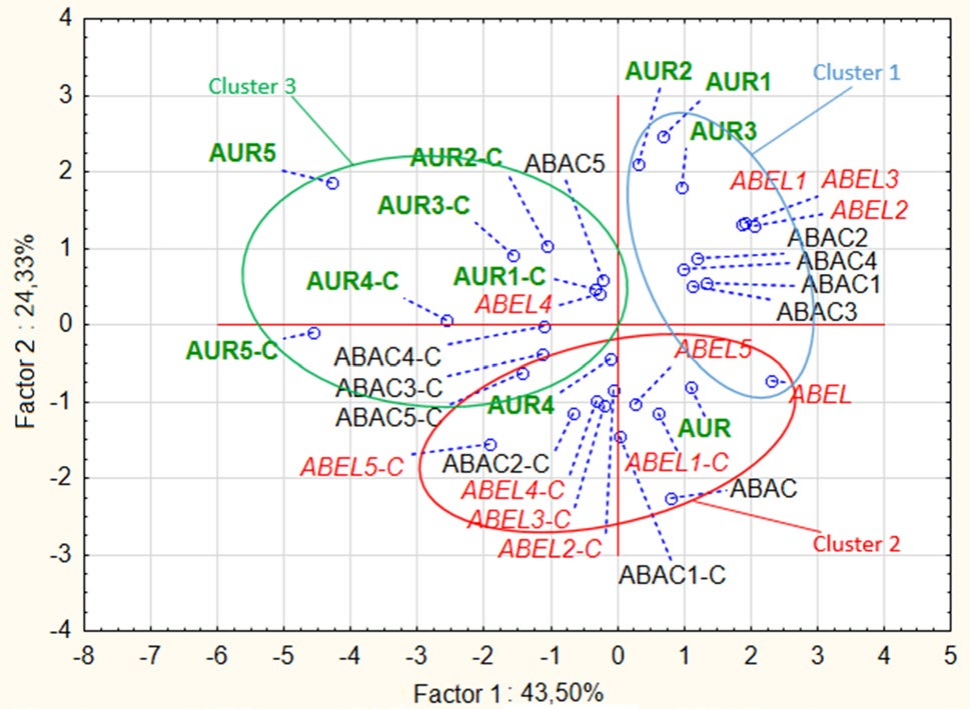
Score plot of principal component analysis of isoflavonoid content in unmalted soybean seeds and soybean malts.

Significant differences in the concentration of various isoflavonoids could be detected between various soybean varieties, drying type and germination time, as well as the fact, whether the soybeans were malted or not. Abaca variety soybeans and malts were characterised with the highest concentration of daidzin among all of the analysed soybeans and soybean malts. In Abelina variety soybeans and malts highest concentration of genistin and glycitein were detected. Aurelina variety soybeans and malts were characterised with highest concentration of genistein. Abaca and Aurelina variety soybeans and malts were also characterised with similar concentration of daidzein and glycitin, higher than concentration of these isoflavonoids detected in Abelina soybeans and malts. However, different concentration of isoflavonoid content in various soybean cultivars is nothing new, as other researchers, analysing large numbers of soybean accession, have shown, that concentration of isoflavonoids in various different soybean cultivars can vary even sevenfold, which is far greater discrepancy than detected in this study^32^. More important result of this study is the data concerning concentration of various isoflavonoids and germination of the soybean seeds. To explain the possible mechanisms, which had an influence on the concentration of isoflavonoids in soybean seeds during germination, it is necessary to describe important distinction between two main groups of the analysed compounds. Daidzein, genistein and glycitein are aglycones with a simple flavan-3-ol skeleton, while daidzin, genistin and glycitin are their derivatives in the form of 7-O-β-D-glucosides, with a molecule of glucose connected to the flavan-3-ol skeleton^3^. It can be seen, that germination has vastly different influence on the concentration of aglycones and 7-O-β-D-glucosides. Generally, malts have lower concentration of 7-O-β-D-glucosides than their ungerminated counterparts. Similar results were detected by Simons et al.^18^ in a study about germinated soybeans challenged by the *Rhizopus* fungi^17^. Additionally, it can be seen that lengthening the germination process results in higher losses of glycitin (i.e. samples germinated for 120 h have lowest concentration of glycitin, while samples germinated for 24 h have the highest). Daidzin concentration in the malts is lower than in unmalted soybeans, but stays on the similar level irrespectively of the germination time. Changes in the level of genistin are less straightforward, as they are lower than in unmalted soybeans, but malts germinated for 24, 48 or 72 h were characterised with lower concentration of this aglycone than malts germinated for 96 h or 120 h, which suggest, that at some point of the germination process, the soybean seeds start synthesizing genistin once again. PCA analysis shows (Fig. 4), that similar phenomenon, influencing concentration of the isoflavonoids during first three days of germination, can be present in soybeans of different varieties, because all malts, which were germinated for up to three days and dried in the lower temperature are present in the first cluster.

**Figure 5:**
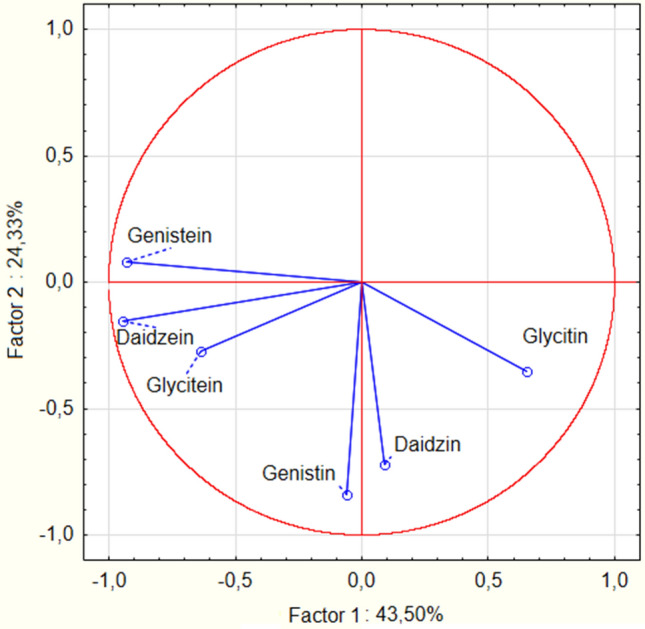
Loading plot of principal component analysis of isoflavonoid content in unmalted soybean seeds and soybean malts.

Additionally, an important difference between the soybean varieties can be seen during the germination time of 96 h and 120 h. In the Abaca variety malts dried in the lower temperature, concentration of daidzin, genistin and glycitin is similar in the samples ABAC4 and ABAC5, while the concentration of daidzein, genistein and glycitein increases during the last 24 h of germination. In the Abelina variety malts dried in lower temperature, during the last 24 h of germination, concentration of daidzin, denistin and glycitein increases, while concentration of daidzein and genistein decreases. In Aurelina variety malts dried in the lower temperature, other changes can be noted, as the concentration of daidzin, genistin and glycitin decreases significantly in the last 24 h of germination, while concentration of daidzein, genistein and glycitein increases. These results suggest, that different soybean varieties might possess different amounts (or activities) of enzymes which are necessary for the conversion of 7-O-β-D-glucosides to aglycones or, possibly, enzymes of the different varieties of soybeans have different optimal temperature of action. Concentration of aglycones undergoes different changes than the concentration of 7-O-β-D-glucosides during the course of malting. Unmalted soybeans have the lowest concentration of daidzein and genistein. Additionally, glycitein is absent in the unmalted soybeans. It can be seen, that concentration of aglycones increases with the lengthening of the germination time—content of aglycones in malts germinated 24 h was lowest (even similar to the concentration in unmalted soybeans in the case of daidzein), while in the malts germinated for 120 h it was the highest. The increase was significant—level of the daidzein content increased over two-fold (from 30.37 to 64.94 ppm) and level of genistein increased almost four-fold (from 28.72 to 105.86 ppm). The specific process of changes of the concentration of isoflavonoids in the soybean malts cannot be precisely determined on the basis of this study, but data gathered in this study, compared with the data from the studies of others scientists can suggest various mechanisms. The decrease of 7-O-β-D-glucosides can possibly be a result of various enzymatic reactions in the hydrated seed, during which the glucose molecule was cleaved out from the 7-O-β-D-glucoside^33^. These results were confirmed by other authors, analysing soybean sprouts, where the concentration of 7-O-β-D-glucosides were decreased to 30–70% of the concentration detected in the ungerminated soybean^30,34^. Due to that fact, the increase in the concentration of aglycones is possibly connected to the reduced concentration of 7-O-β-D-glucosides, because daidzein, genistein and glycitein are one of the products of enzymatic hydrolysis of daidzin, genistin and glycitin^33^. However, the data presented on the Fig. 5 shows, that changes in the concentration of genistein, daidzein and glycitein are more correlated with each other than the changes of genistin, daidzin and glycitin.

It can be seen, that glycitin and glycitein content are negatively correlated, which suggests, that the increasing glycitein content in the longer germinated malts is mainly a result of the transformation of the glycitin present in the soybeans. Therefore, it is also important to note that, while the aglycone concentration increases, the increase is not always on the same level as the decrease of 7-O-β-D-glucosides. However, changes of genistin and daidzein do not correlate with changes in the concentration of genistein and daidzein. This suggests that there are some other products which can transform into 7-O-β-D-glucosides or that various other mechanisms of generation of aglycones can be present in the germinating soybean seed. This data implies, that these various unknown mechanisms of the aglycone generation present in the germinating soybean seeds need to be analysed in the future to expand the knowledge about the viability of production of soy-based products with increases isoflavonoid content. Combined, these data suggest that subsequent study delving into mechanisms of aglycone production in the germinating soybean might give interesting insight into soybean metabolism. Nevertheless, results of this study show, that technique of malting seems like a good tool in the production of various soy products with the increased concentration of aglycones. Last of the analysed factors influencing concentration of isoflavonoids in soybean malts was the method of drying of the germinated seeds, because the drying procedure can be used to create multitude of malts, which differ in aroma, colour, taste, composition and their technological parameters^9^. Soybean malts were dried in two different temperatures: in 50 °C and in controlled temperature programme designed for the production of caramel malts, in which the temperature reached 110 °C. Five out of the six analysed isoflavonoids were detected in the ‘caramel’ type malts in a higher concentration than in malts dried at 50 °C, while the sixth isoflavonoids (glycitin) was detected at the similar level. These results strongly suggest, that the higher temperature during the drying has significant influence on the increase of isoflavonoids in the soybean malts. In the research of de la Bastida et al.^35^ about storage and heat treatment on the concentration of isoflavonoids in soybean-based beverages, a slight increase in the concentration of daidzin, daidzein, genistin and genistein was noted during the heat treatment. On the contrary, previous research by Stintzing, Hoffmann and Carle^36^ shows, that daidzein, glycitein and genistein are reduced due to the heat treatment. It is, however, necessary to mention, that during the experiment of Stintzing et al. higher temperature (150 °C) was used for a prolonged amount of time (up to 7 h) and, nevertheless, the isoflavone content of the soybeans remained almost constant for 3 h of heat treatment. As it can be seen in Fig. 4, malts of the ‘caramel’ type were grouped in two different clusters, which suggests, that varieties of soybeans respond in a different way to drying in the higher temperature. ‘Caramel’ malts from Abelina variety were grouped together, with unmalted samples ABEL, ABAC and AUR, while most of the the caramel malts from varieties Abaca and Aurelina shared cluster 3 together. As can be seen on the Figs. 1, 2 and 3, changes in the concentration of isoflavonoids in the Abelina variety malts dried in higher temperature are less variable than the concentration of isoflavonoids in the Abaca and Aurelina, as most of the compounds (with exception of glycitin) remain at similar level in the Abelina caramel-type malts, with large increase only during the last 24–48 h of germination. In the caramel-type malts of Abaca and Aurelina variety, concentration of various isoflavonoids varies more significantly with the changes of the germination time. This results might indicate, that possibly enzymes responsible for the changes in the isoflavonoid content in the soybean malts in the Aurelina are less temperature-susceptible that in Abelina or Abaca, but this assumption has to be investigated further in a more detailed study in the duture. The data gathered in this study strongly suggests that various kinds of heat treatment are beneficial in increasing the isoflavonoid content, while some of the heat treatments are detrimental to acquiring high isoflavonoid level. Future studies concentrating on this particular aspect of soybean malt production, with the goal of optimisation of drying process might be of interest to the scientific community. Principal component analysis (PCA) of all the factors analysed in this study, presented in the Fig. 4 (loading plot) and Fig. 5 (score plot) helps to visualise the effects of malting procedure on various samples and isoflavonoids analysed in this study. Results acquired in this study show, that simple procedure of malting could be used as a reliable method in the modification of isoflavonoid content of soybeans and might open new venues of the food technology connected with designing high isoflavonoid food products in the future.

## Conclusions

This study shows that malting technology can be used to increase concentration of various isoflavonoid aglycones in the soybean seeds. Data gathered during the research additionally shows, that increasing germination time can also influence decrease of the 7-O-β-D-glucosides (daidzin, genisitin and glycitin), as well as increase of the aglycones (daidzein, genistein and glycitein). Increasing the germination time during malting procedure results in increase of most isoflavonoids in the soybean malts analysed in this study. Furthermore, acquired data shows, that seed drying in the higher temperatures with recirculation of water in the dryer during the first stages of the drying results in malts with higher concentration of 7-O-β-D-glucosides and aglycones alike.

## Data Availability

The datasets generated and analysed during the current study are available in the figshare repository available at https://doi.org/10.6084/m9.figshare.24533161.v1.

## References

[1] Agyei D (2015). Bioactive proteins and peptides from soybeans. Recent Pat. Food Nutr. Agric..

[2] Rebholz CM (2013). Effect of soybean protein on novel cardiovascular disease risk factors: a randomized controlled trial. Eur. J. Clin. Nutr..

[3] Dastmalchi M, Dhaubhadel S, Jetter R (2014). Soybean seed isoflavonoids: Biosynthesis and regulation. Phytochemicals-Biosynthesis, Function and Application.

[4] Miadoková E (2009). Isoflavonoids–an overview of their biological activities and potential health benefits. Interdiscip. Toxicol..

[5] McCue P, Shetty K (2004). Health benefits of soy isoflavonoids and strategies for enhancement: A review. Crit. Rev. Food Sci..

[6] Thompson MJ (2010). Isoflavones: biosynthesis, occurrence, and health effects.

[7] Bi W (2022). Metabonomics analysis of flavonoids in seeds and sprouts of two Chinese soybean cultivars. Sci. Rep..

[8] Miyahira RF, Antunes AEC (2021). Bacteriological safety of sprouts: A brief review. Int. J. Food Microbiol..

[9] Kunze W (2019). Brewing & Malting.

[10] Gasiński A, Kawa-Rygielska J (2022). Mashing quality and nutritional content of lentil and bean malts. LWT.

[11] Gasiński A, Kawa-Rygielska J, Mikulski D, Kłosowski G (2022). Changes in the raffinose family oligosaccharides content in the lentil and common bean seeds during malting and mashing processes. Sci. Rep..

[12] Mäkinen OE, Arendt EK (2015). Nonbrewing applications of malted cereals, pseudocereals, and legumes: A review. J. Am. Soc. Brew. Chem..

[13] Banusha S, Vasantharuba S (2013). Effect of malting on nutritional contents of finger millet and mung bean. Am. Eurasian J. Agric. Environ. Sci..

[14] Egli I, Davidsson L, Juillerat MA, Barclay D, Hurrell RF (2002). The influence of soaking and germination on the phytase activity and phytic acid content of grains and seeds potentially useful for complementary feedin. J. Food Sci..

[15] Nnanna IA, Phillips RD (1988). Changes in oligosaccharide content, enzyme activities and dry matter during controlled germination of cowpeas (*Vigna unguiculata*). J. Food Sci..

[16] Rao PU, Belavady B (1978). Oligosaccharides in pulses: varietal differences and effects of cooking and germination. J. Agric. Food Chem.

[17] Miller YY, Kiseleva TF, Arysheva IV (2021). Forming soy malt quality with organic growth promoters. Food Process. Tech. Technol..

[18] Simons R, Vincken JP, Roidos N, Bovee TF, van Iersel M, Verbruggen MA, Gruppen H (2011). Increasing soy isoflavonoid content and diversity by simultaneous malting and challenging by a fungus to modulate estrogenicity. J. Agric. Food Chem..

[19] Barnes S, Prasain J, D'Alessandro T, Arabshahi A, Botting N, Lila MA, Jackson G, Janle EM, Weaver CM (2011). The metabolism and analysis of isoflavones and other dietary polyphenols in foods and biological systems. Food Funct..

[20] Villares A, Rostagno MA, García-Lafuente A, Guillamón E, Martínez JA (2011). Content and profile of isoflavones in soy-based foods as a function of the production process. Food Bioproc Tech..

[21] Kao TH, Lu YF, Hsieh HC, Chen BH (2004). Stability of isoflavone glucosides during processing of soymilk and tofu. Food Res. Int..

[22] Prabhakaran MP, Perera CO, Valiyaveettil S (2006). Effect of different coagulants on the isoflavone levels and physical properties of prepared firm tofu. Food Chem..

[23] Yin LJ, Li LT, Liu H, Saito M, Tatsumi E (2005). Effects of fermentation temperature on the content and composition of isoflavones and beta-glucosidase activity in sufu. Biosci. Biotechnol. Biochem..

[24] Wei QK, Chen TR, Chen JT (2008). Use of *Bacillus subtilis* to enrich isoflavone aglycones in fermented natto. J. Sci. Food Agric..

[25] Chiou RY-Y, Cheng S-L (2001). Isoflavone transformation during soybean koji preparation and subsequent miso fermentation supplemented with ethanol and NaCl. J. Agric and Food Chem.

[26] Nakajima N, Nozaki N, Ishihara K, Ishikawa A, Tsuji H (2005). Analysis of Isoflavone content in tempeh, a fermented soybean, and preparation of a new isoflavone-enriched tempeh. J. Biosci. Bioeng..

[27] Chang TS, Ding HY, Tai SSK, Wu CY (2007). Metabolism of the soy isoflavones daidzein and genistein by fungi used in the preparation of various fermented soybean foods. Biosci. Biotechnol. Biochem..

[28] Genovese MI, Lajolo FM (2002). Isoflavones in soy-based foods consumed in Brazil: Levels, distribution, and estimated intake. J. Agric. Food Chem.

[29] Gasiński A, Kawa-Rygielska J, Spychaj R, Opiela E, Sowiński J (2023). Production of gluten-free beer brewing from sorghum malts mashed without external enzyme preparations. J. Cereal Sci..

[30] Wang SY (2022). Occurrence of isoflavones in soybean sprouts and strategies to enhance their content: A review. J. Food Sci..

[31] Zdzieblo, A., & Reuter, W. M. *Analysis of Isoflavones in Soy Products by UHPLC with UV Detection* (Perkin Elmer Application Note) (2015). Accessed 6 Oct 2022; https://resources.perkinelmer.com/lab-solutions/resources/docs/app-analysis-of-isoflavones-in-soy-products-by-uhplc.pdf.

[32] Azam M (2020). Seed isoflavone profiling of 1168 soybean accessions from major growing ecoregions in China. Food Res. Int..

[33] Suzuki H (2006). An isoflavone conjugate-hydrolyzing β-glucosidase from the roots of soybean (*Glycine max*) seedlings: Purification, gene cloning, phylogenetics, and cellular localization. J. Biol. Chem..

[34] Huang X, Cai W, Xu B (2014). Kinetic changes of nutrients and antioxidant capacities of germinated soybean (*Glycine max* L.) and mung bean (*Vigna radiata* L.) with germination time. Food Chem..

[35] de la Bastida AR (2022). Effect of storage and heat treatment on the levels of bioactive flavonoids produced in fermented soy beverages. LWT.

[36] Stintzing FC, Hoffmann M, Carle R (2006). Thermal degradation kinetics of isoflavone aglycones from soy and red clover. Mol. Nutr. Food Res..

